# A Question of Rights and Privileges

**Published:** 1911-03

**Authors:** George L. Servoss

**Affiliations:** Fairview, Nevada


For Texas Medical Journal.

### A Question of Rights and Privileges.

BY GEORGE L. SERVOSS, M. D., FAIRVIEW, NEVADA.

There is not a State in the Union which does not have upon its statute books certain laws governing the practice of medicine. These laws prescribe certain qualifications required entitling the doctor the privilege to practice his chosen profession, but do not, in a single instance, require him to follow any particular school, or sect, of medicine, providing he has proven himself competent, in the eyes of the board of examination, to follow his vocation in such a manner as not to become a menace to those upon whom he is called to attend. These laws do not, for constitutional reasons, dictate the remedies in individual cases, or the source of supply of such remedies. In fact, it is inferred by the statutes in question that, when a doctor has proven himself competent to practice medicine, it is shown that he not only knows the application of the remedies indicated in certain cases, but likewise knows the remedies themselves, and consequently is able to handle and dispense such remedies intelligently. If such were not the case, no certificate entitling him to practice medicine would have been given him by the board before which he may have appeared.

It has been said that the doctor, during his medical course, has not been given a thorough course in pharmacology and pharmacy, and, consequently, can not have a sufficiently comprehensive knowledge of drugs to enable him to either compound such drugs or dispense them intelligently. It has been said that the average doctor has little, if any, knowledge of the physical appearance or condition of the agents employed by him in the treatment of disease; in fact, that he does not know the tools of his vocation. In consequence of this contention, it is further contended that he should not be allowed the privilege of dispensing.

It may be true that the doctor who writes prescriptions at all times does not come sufficiently in contact with the agents employed by him to be conversant with their physical characteristics, or combining powers, and that, because of this, he is not sufficiently well informed to compound the mixtures indicated in the cases under his observation. On the other hand, the dispenser, through his daily contact with the agents used in his practice, not only becomes thoroughly conversant with their therapeutic actions and applications, but with all else connected with them. The dispenser, through compounding, discovers many little pharmaceutical points which are, as a rule, unknown to the prescription writer. Being a compounder, as well as an applier of remedial agents, the dispenser is obliged to study the physical peculiarities of drugs, in that he may not be the loser through faulty compounding. The dispenser, if he would meet with success, is obliged to follow the same rules as are laid down for the druggist regarding the proper handling of drugs and chemicals. The druggist obtains these rules from the U. S. P., N. F., dispensatories and other works of reference, which are really recipe books, the result of previous experiments. The dispensing doctor has these same books at hand and, by following the working formulas and technic laid down in each individual case, is able to compound as well as is the druggist. Such being the case, the dispenser, providing he follows the rules laid down by the authorities, is entitled to the same rights and privileges as is the pharmacist, following like rules.

In the majority of instances, new remedies and new applications of older ones have been introduced by the doctor, and not the pharmacist. In fact, there are very few instances on record where the pharmacist has taken the initiative in such action. Such being the case, is not the doctor capable of dispensing and applying such remedies without having first to write a prescription ordering them? Would not a law compelling the doctor to write a prescription for such remedies interfere with his rights and privileges? Ehrlich, who is a doctor, discovered, dispensed and applied "606," and all as a doctor. Would it have been just to him to have been required to ask a pharmacist to do his compounding for him? Would he have been assured that the resulting chemical would have been absolutely the same as that made by him? Any law compelling him to have gone outside his own laboratory would have interfered with his rights and privileges as a doctor and an investigator. If this is true with Ehrlich, is it not likewise true with the vast army of dispensers?

It has been contended that the doctor employs inferior drugs in dispensing and that he buys his supplies of manufacturers making notoriously inferior goods. How can this be when all the manufacturers of pharmaceuticals are obliged to comply with certain statutes as regards the purity and standard of their products? Should the doctor be condemned because of the fact that the manufacturer furnishes him with goods below standard? Would not such condemnation be considered as an interference with his personal rights and privileges? Would it not be better that restrictions be placed upon the manufacturers and that they be compelled, at all times, to furnish both pharmacists and doctors with only reputable products? It is said that the pharmacist is better equipped to examine and test his goods, than is the doctor, but in how many instances are any such tests made? The doctor may not make analyses of the products purchased by him, but in the majority of instances discovers, through clinical application, whether or not the goods furnished him are therapeutically active. If they are found wanting, the doctor either complains to his base of supplies, or make his purchases elsewhere. *It is absolutely necessary, if he desires success to follow his efforts, be he a dispenser or prescription writer, that the doctor be furnished with agents, active in every instance and of a recognized standard of*

*strength and quality. Every doctor recognizes this to be an absolute truth* and, as a rule, *the dispenser is very particular regarding the quality of goods furnished him.* He recognizes the fact that, if he carries a stock of inactive drugs and other therapeutic agents, it will interfere to an alarming extent with his success as a practician. He knows to a certainty the derivation of his supplies and whether they are up to standard or not. Would not laws, making dispensing a misdemeanor, interfere with the personal rights and privileges of the doctor? Would such laws give the patient greater assurance of obtaining any better treatment, in so far as remedies might be concerned? Is it not possible that the pharmacist, like the doctor, might be furnished with drugs far below standard? This has been proven true in more instances than one, as has been shown by recent reports of investigations.

In spite of all that has been said and done by those who hold the dispenser in disdain, he will not be legislated out of existence, as he possesses, and will continue to possess, inalienable rights which can not be taken from him through legislative action. If the public is to be protected, and it seems that the public is the main consideration in this controversy, let not only the dispensing doctor be under the eye of the law, but the pharmacist and manufacturing pharmacist, in that both the dispenser and druggist be furnished with only reputable goods. The laws in effect at this time are sufficiently rigid and should be enforced and if such enforcement is followed there will be no reason for questioning the goods handled by either the dispenser or druggist. Let there be an inspection of the stocks of both the doctors and druggists, and such goods as may be found below par be thrown out. Then let there be an inspection of the factories making such goods, in that they may be required to manufacture goods which will meet with the required standards at all times. It was a notorious fact that, prior to the going into effect of the Pure Food and Drugs Law, there were numerous manufacturers who made and marketed inferior drug products, but, even at that time, such products failed to give anticipated results and lacked a general popularity with the majority of dispensers. *Some druggists bought goods from these houses, and filled prescriptions from them, regardless of the fact that they were known to be lower in price than like goods, in so far as formula might be concerned, made by reputable houses, and, because of this, probably inferior in quality.* It did not take the dispenser long to discover the inferiority of such goods, and, because of their inactivity, they were discarded. On the other hand, the prescription writer, not coming in personal contact with the tools of his profession, frequently laid the inactivity of the drugs of this sort to other causes, and *the goods continued to be employed,* despite their inferiority and frequent inactivity. In instances of this character the rights of the patient were interfered with, as he was not furnished with remedies of sufficient potency to bring him anticipated relief.

In the contention that the doctor should not be allowed to dispense, because of his lack of drug knowledge, in so far as compounding is concerned, an exception is to be made in emergencies. Is such an exception consistent? If he is unable to dispense intelligently in all instances, how can he do so in emergencies? Emergencies frequently call for compounds and, if the doctor can not make them for all sorts and characters of cases, how can he be expected to make them for emergencies? If he has a right to dispense in such cases, has he not as much right to dispense in all cases? Would not this single exception prove that anti-dispensing legislation, as a whole, would be faulty and an interference with the rights and privileges of the doctor to follow his vocation as he desired, and that such a law would be unconstitutional, because of this one factor?

The doctor, through the rights invested in him by the school from which he graduates and later through the certificate of the examining board before which he may have appeared, entitles him to practice medicine as he may deem proper, and so long as he employs his drugs and other agents intelligently in the meeting of indications presenting, none can gainsay him these rights. If he does not use drugs which are of standard quality, and it is shown that he follows such a course knowingly, let him come under the activity of the laws governing such cases and be prosecuted for employing inferior goods, but if he does employ standard goods which correspond in every particular with the legal requirements, and is known to apply such agents intelligently he should be allowed to practice medicine as he sees fit, be it as a dispenser or a prescription writer. Let there be a rigid inspection of the manufacturing plants making pharmaceuticals and of the retail shops handling such goods, and the laboratories of the dispensing doctors as well, and let all conform to the recognized standards, and let there be no goods sold by any of the handlers of drugs which do not conform in all ways to the recognized standards of quality. If such a course is pursued, not only will the doctor be assured of his rights, but the patient as well, and it is the latter who should be considered at all times, as his are the paramount rights in the question.

